# Supplementary material for: Pool PaRTI: a PageRank-based pooling method for identifying critical residues and enhancing protein sequence representations
Source: Bioinformatics. 2025 Jun 2;41(6):btaf330. doi: 10.1093/bioinformatics/btaf330 (PMC12203179; doi:10.1093/bioinformatics/btaf330)
Supplement: btaf330_Supplementary_Data [file btaf330_supplementary_data.pdf]

Supplementary Material for
Pool PaRTI: A PageRank-Based Pooling Method for Identifying Critical Residues and Enhancing Protein Sequence Representations

Alp Tartici
Stanford University
tartici@stanford.edu

Gowri Nayar
Stanford University
gnayar@stanford.edu

Russ B Altman
Stanford University
rbaltman@stanford.edu

1 Supplemental Explanation

1.1 Empirical analysis of Pool PaRTI runtime scaling

To formally assess the empirical scaling of runtime for the Pool PaRTI algorithm, we executed the algorithm on 25 protein sequences, repeating each run 15 times. We then applied a logarithmic transformation to both the runtime data and the corresponding sequence lengths. A linear regression model was fitted to the log-transformed data, yielding a slope of 2.068, which aligns with our theoretical reasoning in Section **??**. This slope indicates the exponent in the power-law relationship between the algorithm's runtime and the input size, providing a quantitative measure of the algorithm's scaling behavior with respect to input size.

1.2 Deep Learning Models and Configurations

1.2.1 Subcellular Localization Prediction Task

Description of the model

- 2 x (linear layer + leaky ReLU + dropout)
- Linear layer
- Input residual connection
- Sigmoid

Fixed configurations

- Initial learning rate: 0.01
- Max number of epochs: 1000
- Early stopping patience epochs: 50
- Optimizer: AdamW

Preprint. Under review.

- Learning rate scheduler: ReduceLROnPlateau
- Optimizer learning rate patience epochs: 10
- Learning rate reduction ratio: 0.1
- Weight Initialization: Xavier normal
- Random seed: 42
- Gradient clip value: 5.0
- batch size: 64

Hyperparameter optimization space

- Weight decay: [0.01, 0.1, 0.2]
- Dropout rate: [0.15, 0.25]
- Slope of Leaky ReLU: [0.01, 0.1]
- Exponent for imbalance penalty taming: [0.75, 1, 1.25]

1.2.2 Protein-Protein Interaction Prediction Task

Description of the model

- 1 x (linear layer + batch norm + leaky ReLU + dropout)
- 1 x (linear layer + linearly transformed residual connection + leaky ReLU)
- Linear layer

Fixed configurations

- Hidden dimensions: 1024
- Max number of epochs: 40
- Early stopping patience: 9
- Optimizer: AdamW
- Learning rate scheduler: ReduceLROnPlateau
- Optimizer learning rate patience epochs: 4
- Random seed: 42
- Weight initialization: Kaiming normal
- Gradient clip max value: 2.0
- batch size: 32

Hyperparameter optimization space

- Initial learning rate: [0.001, 0.01]
- Weight decay: [0.01, 0.1, 0.2]
- Dropout: [0.05, 0.15, 0.25]
- Reduction in learning rate ratio: [0.2, 0.5]
- Leaky ReLU slope: [0.01, 0.1, 0.2]

1.3 Data and code availability

Our PyTorch implementation of Pool PaRTI and the experimental task models are available at `https://github.com/Helix-Research-Lab/Pool_PaRTI.git`. The Pool PaRTI residue importance values for all human proteins on UniProt are available at `https://zenodo.org/records/15036725` for ESM2 and protBERT. The data we used in these tasks are as follows: For the subcellular localization prediction task, we used the data annotation and splits provided by Thumuluri et al [1]. For the protein-protein interaction prediction tasks, we used the data annotation and gold standard split provided by Bernett et al. [2]. For the enzyme classification task, we used the gold standard ECPred40 dataset created by Buton et al. [3]. We obtained the SCOP labels from the SCOP database [4, 5] with API access.

1.4 Compute resources for experiments

We ran all computational experiments and pooling algorithms on Tesla V100-SXM2-16GB GPUs housed in the internal Sherlock cluster. For each task, the hyperparameter optimization experiments were limited to two days on GPU. On the same GPUs, we conducted unpublished preliminary experiments in developing the Pool PaRTI algorithm. We generated the precomputed ESM2 token embeddings on NVIDIA A100-PCIE-40GB GPUs, also housed in the internal Sherlock cluster. Token embedding generation took 70 GPU hours through the ESM2 650M model and 50 hours through protBERT model. We have computed token embeddings once for each sequence and performed several different pooling operations on the precomputed token embeddings before feeding the sequence embeddings as inputs to the respective models.

References

[1] Vineet Thumuluri et al. "DeepLoc 2.0: multi-label subcellular localization prediction using protein language models". In: *Nucleic Acids Research* 50 (W1 July 5, 2022), W228–W234. ISSN: 0305-1048, 1362-4962. DOI: 10.1093/nar/gkac278. URL: https://academic.oup.com/nar/article/50/W1/W228/6576357 (visited on 01/13/2024).

[2] Judith Bernett, David B Blumenthal, and Markus List. "Cracking the black box of deep sequence-based protein–protein interaction prediction". In: *Briefings in Bioinformatics* 25.2 (Jan. 22, 2024), bbae076. ISSN: 1467-5463, 1477-4054. DOI: 10.1093/bib/bbae076. URL: https://academic.oup.com/bib/article/doi/10.1093/bib/bbae076/7621029 (visited on 04/15/2024).

[3] Nicolas Buton, François Coste, and Yann Le Cunff. "Predicting enzymatic function of protein sequences with attention". In: *Bioinformatics* 39.10 (Oct. 3, 2023). Ed. by Alfonso Valencia, btad620. ISSN: 1367-4811. DOI: 10.1093/bioinformatics/btad620. URL: https://academic.oup.com/bioinformatics/article/doi/10.1093/bioinformatics/btad620/7329097 (visited on 01/22/2024).

[4] Antonina Andreeva et al. "The SCOP database in 2020: expanded classification of representative family and superfamily domains of known protein structures". In: *Nucleic Acids Research* 48 (D1 Jan. 8, 2020), pp. D376–D382. ISSN: 0305-1048, 1362-4962. DOI: 10.1093/nar/gkz1064. URL: https://academic.oup.com/nar/article/48/D1/D376/5625529 (visited on 09/27/2024).

[5] Antonina Andreeva et al. "SCOP2 prototype: a new approach to protein structure mining". In: *Nucleic Acids Research* 42 (D1 Jan. 2014), pp. D310–D314. ISSN: 0305-1048, 1362-4962. DOI: 10.1093/nar/gkt1242. URL: https://academic.oup.com/nar/article-lookup/doi/10.1093/nar/gkt1242 (visited on 09/27/2024).
